# The psycho-lexical structure of sports culture

**DOI:** 10.3389/fpsyg.2022.894694

**Published:** 2022-10-05

**Authors:** Yuanbing Guo, Na Ye, Xiaobin Hong, Wenjing Wang

**Affiliations:** ^1^College of Sports Science and Technology, Wuhan Sports University, Wuhan, China; ^2^Hubei Provincial Key Laboratory of Sports Training Monitoring, Wuhan Sports University, Wuhan, China; ^3^Department of Applied Psychology, Wuhan Sports University, Wuhan, China

**Keywords:** sports culture, psycho-lexical hypothesis, psychological structure, factor analysis, psychological adjectives

## Abstract

The psycho-lexical approach can be effectively used to explore the structure of sports culture. Based on a lexical list of adjective vocabulary reflecting sports culture and through an item analysis, 87 discriminating objectives were selected representing sports culture.

**Objective:**

To explore the structure of sports culture from the objectives lexicons.

**Methods:**

Item analysis and factor analysis were adopted to abstract the structure of sports culture.

**Results:**

Through a principal component analysis, a structure of six factors including extroversion-activity, diligence-progression, experience, independence-excellence, enjoyment, and body culture was extracted. Through a second-order factor analysis, a psychological structure of sports culture consisting of six dimensions and 12 factors was extracted, and result of the reliability analysis revealed good Cronbach α coefficient and test–retest reliability coefficient.

**Conclusion:**

The psycho-lexical structure of sports culture can be used to understand structure of sports culture.

## Introduction

Sport is a cultural phenomenon on a natural biological basis (Mcpherson et al., 1989). In the era of “sports society,” sports culture, divided into specific sports culture and sportification in general (Grupe, 1994), is perceived as an expression of modernism. With the turn to cultural philosophy, sports culture has become an inspiring topic in the field of sports study. Despite this substantial uptick in interest, however, the rapidly growing literature on sports culture suffers from two notable limitations. First, there is no clear consensus about precisely what the structure of sports culture is (Luschen, 1967; Krawczyk, 1980; Rodesiler, 2021). Relatedly, the broad definition leads to too much research on generality and not enough research on specificity (Lahire, 2008).In the view of this, new theories and specific frameworks must be adopted to transcend existing disciplinary boundaries and to reshape research on sports culture (Takamatsu, 1987; Bilohur and Andriukaitiene, 2020).

### Structure of sports culture

A few empirical studies focusing on the structure of sports culture explored the differences and sportification. These studies were mainly carried out on three research aspects. First, on the value and spirit dimension of sports culture. For example, Lee et al. (2000) developed the Youth Sport Values Questionnaire. Obasa and Borry (2019) analyzed the multivalence of the concept of spirit of sport. The more adequate study among them is the personality structure of sports events (Lee and Cho, 2012; De Vries, 2020). Secondly, on the direct exploring of sports culture’s structure. For example, Kaiser et al. (2009) used Structure Dimensional Analysis (SDA) as an innovative approach to understand the representation of culture-related knowledge in sport organizations. Culpepper and Killion (2015) applied the symbolic analysis to identify the general cultural categories of sports (i.e., movement forms, rules, and values). Thirdly, on the sub-sector of sports culture. For example, regional features of sports culture (Adedeji, 1979; Hargreaves, 1997; Sibaja, 2015), psychometric properties of sport participation (Beaton et al., 2011; Casper et al., 2011) and the structural properties of sport teams culture (Kao and Cheng, 2005; McEwan, 2017) were studied. However, previous studies have not been sufficient to reveal the structure of sports culture, which identifies the cultural function of sport and legitimizes it as a social institution (Kono, 1997). Therefore, there is a need to further investigate the conceptual system, measurement tools, and structural features of sports culture.

### The lexical paradigm

The psycho-lexical approach, which uses language to study culturally loaded words, is based on the assumption that the most important individual difference have been encoded into the natural language (Allport and Odbert, 1936). Based on studies that use the lexical paradigm, by following a standard procedure to extract the most common trait-related words from sufficiently large lexicon, and by asking respondents to indicate the extent to which these words represent their, or an acquaintance’s–personality, researchers across the world have been able to arrive at a near consensus about the main dimensions of personality (Goldberg, 1981). The lexical approach has been applied to several psychology domains, such as values (De Raad et al., 2017; Wilkowski et al., 2021), emotions (Macheta and Gorbaniuk, 2020), interpersonal interactions (Strachan et al., 2009), and communication styles (Labasse, 2001; De Vries et al., 2009), but it has rarely been applied to the sport psychology and sports culture domain. As noted above, such a lexical study may provide provides a way to explore the structure of sports culture.

### The current study

The aim of our study is to uncover the psycho-lexical structure of sports culture. The lexical study on social scientific domain was conducted in three phases. In the first phrase, a preliminary selection of adjectives that pertained to sports culture was made. In the second phrase, a further reduction of the list of adjectives was made base on a panel of experts. In the third phrase, self-ratings were obtained on the list of adjectives selected. Guo and Qi (2017), by using the psychological vocabulary method, a total of 372 college students were investigated, and a total of 694 typical adjectives as carriers representing the three levels of people, things and incidents in sports culture were collected. After synonym classification and frequency sorting, 87 adjectives representing sports culture were obtained which could provide a basis for the next step to confirm the structure of sports culture. With regard to this research, the Chinese psycho-lexical project in the sports domain is discussed in the present study. The extracted psychological structure of sports culture would possibly pave the way for further empirical studies.

## Materials and methods

### Participants

The sample 1 (*n* = 128 college students, 72 males and 56 females, *M*_age_ = 19.11, SD = 1.54) for item analysis were randomly selected from a university in Wuhan, China.

The sample 2 (*n* = 505 college students, 298 males and 207 females, *M*_age_ = 19.20, SD = 1.62) for factor analysis were randomly selected from two universities in Wuhan, China.

The sample 3 (*n* = 223 college students, 115 males and 107 females, *M*_age_ = 19.34, SD = 1.52) for test–retest reliability analysis were randomly selected from a university in Wuhan, China.

The trained graduate students of psychology explained the requirements of the survey using standard instructions emphasizing the authenticity, independence, and integrity of all answers.

### Materials and procedure

#### Materials

“Survey of sports culture carriers” phase: by adopting survey of sports culture’s carrier, participants were asked to give specific examples of sports culture carriers from three categories: people, events and objects of sports.

“Collection of sports culture adjectives” phase: participants were asked to anthropomorphize the listed sports culture carriers and then describe their qualities in free association, resulting in 694 adjectives.

“Lexical screening of sports culture adjectives” phase: The more frequently the vocabulary is used, the more important the feature being described is. Therefore, the third step of vocabulary sorting is to perform frequency calculation on the obtained 124 vocabularies. After removing the bottom 27% low-frequency words in the lexical frequency ranking, and confirmed by two experts in linguistics, 91 basic vocabularies were obtained.

“Measurement of the Significance of Sports Culture Adjectives” phase: The significance mean (*M*) and standard deviation (SD) of the 91 adjectives were calculated. The overall score is distributed in negative skewness, which indicated that the respondents have a good understanding of the meaning of these adjectives. After excluding the four adjectives with low meaning and too large standard deviation: bullying, elitist, triumphant, and restrained, the final 87 adjectives were obtained (Guo and Qi, 2017).

#### Procedure

The 87 selected adjectives were first subjected to an Item Analysis. Factor Analysis and Reliability Analysis were then performed on those with significant discriminability.

### Data processing

According to the research requirements,SPSS21.0 software was employed to record data and for data processing.

## Results

### Item analysis

To test the feasibility of revealing sports culture structures from the selected 87 adjectives, each adjective was first subjected to an item analysis. The critical ratio (CR) was employed as an index for item discrimination analysis. According to the t-test results, 10 adjectives (energetic, harmonious, stable, attentive, friendly, imaginative, promising, arrogant, conceited, and self-reliant) with significance levels of greater than 0.01 were eliminated. Finally, 77 adjectives with significant discriminability were obtained (Table 1).

**Table 1:** Results of the item critical ratio (CR) analysis.

Adjective	CR	Adjective	CR	Adjective	CR	Adjective	CR
Indomitable	5.451	Extroverted	3.864	Forthright	4.432	Interesting	5.224
Fond of stimulation	4.044	Excited	4.657	Disciplined	5.233	Insightful	5.245
Flexible	4.667	Masculine	5.278	Optimistic	5.742	Ambitious	5.338
Independent	5.051	Undeterred	5.148	Courageous	4.621	Opinionated	5.217
Bold	5.224	Person of character	4.763	Self-motivated	5.545	Active	5.774
Tolerant	5.330	Aspiring	5.541	Team-spirited	5.266	Thrill-seeking	5.363
Lively	5.374	Persistent	5.062	Advanced	5.364	Eager to excel	4.952
Persevering	5.466	Sane	5.173	Joyful	4.775	Continuously improving	5.710
Healthy	4.702	Dedicated	5.253	Tolerant	5.048	Seeking to better oneself	4.603
Magnanimous	5.370	Tireless	4.715	social	4.742	Fun loving	5.242
Striving for excellence	4.760	Confident	4.836	Valuing friendship	4.861	Tempered	5.035
Hard-working	5.374	Articulate	4.734	Agile	5.242	Enterprising	4.604
Cheerful	5.733	Passionate	5.472	Adaptive	5.621	Good-hearted	5.617
Pioneering	5.151	Impulsive	5.281	Disciplined	5.413	Lively	5.439
Aspiring	5.377	Generous	5.333	Frank and outspoken	5.135	Open	5.742
Muscular	4.266	Casual	5.059	Loyal to friends	5.477	Relaxed	5.374
Powerful	5.415	Focused	4.223	Perfectionistic	5.484	Respectful of the profession	5.498
Vivacious	4.632	Willing to help others	5.207	Pragmatic	4.696		
Comfortable	5.070	Forgiving	5.118	Open-minded	4.642		
Fortitude	5.400	Talkative	5.334	Straightforward	5.120		

### Principal component analysis

Based on the item analysis results, the 77 remaining items were subjected to Bartlett’s test, which generated a value 2460.76 (*p*<0.01) and a KMO of 0.89. A principal component analysis was performed on the 77 adjectives. First, items with loads of less than 0.3 were eliminated, including muscular, tempered, disciplined, pragmatic, insightful, and fond of stimulation. Second, items with multiple comparable loads were removed, including social, healthy, open-minded, ambitious, and enterprising. The remaining items could be explained by six main factors with an explained variance of 57.69%. As shown in Table 2, 17 items were identified as factor *F*1 (extroversion-activity), 15 items were identified as factor *F*2 (diligence-progression), 12 items were identified as factor *F*3 (experience), ten items were identified as factor *F*4 (independence-excellence), five items were identified as factor *F*5 (enjoymen)t, and five items were identified as factor *F*6 (body culture),and corresponding explained variances were measured as 12.90, 11.64, 10.15, 9.27, 7.01, and 6.69%, respectively.

**Table 2:** Factor load matrix of psychological structures of sports culture.

Adjective	*F*1	*F*2	*F*3	*F*4	*F*5	*F*6
Extroverted	0.681					
Talkative	0.652					
Impulsive	0.669					
Excited	0.625					
Cheerful	0.687					
Optimistic	0.655					
Open-minded	0.597					
Lively	0.583					
Interesting	0.542					
Good-hearted	0.687					
Passionate	0.660					
Bold	0.646					
Courageous	0.657					
Magnanimous	0.542					
Forthright	0.586					
Open	0.610					
Frank and outspoken	0.497					
Tireless		0.586				
Respectful of the profession		0.714				
Persistent		0.661				
Hard-working		0.642				
Dedicated		0.538				
Undeterred		0.703				
Persevering		0.539				
Active		0.448				
pioneering		0.676				
Eager to excel		0.512				
Indomitable		0.467				
Tolerant		0.541				
Valiant		0.643				
Aspiring		0.612				
fortitude		0.638				
Forgiving			0.569			
Sane			0.554			
Flexible			0.571			
Self-motivated			0.657			
Adaptive			0.724			
Team-spirited			0.671			
Articulate			0.577			
Valuing friendship			0.592			
Loyal to friends			0.661			
Willing to help others			0.617			
Willing to accept challenges			0.695			
Ambitious			0.656			
Independent				0.539		
Confident				0.548		
Focused				0.688		
Opinionated				0.519		
Person of character				0.549		
Perfectionist				0.591		
Continuously improving				0.583		
Advanced				0.712		
Striving for excellence				0.567		
Seeking to better oneself				0.601		
Casual					0.546	
Relaxed					0.560	
Fun loving					0.705	
Joyful					0.694	
Comfortable					0.612	
Masculine						0.643
Agile						0.661
Powerful						0.597
Strong						0.604
Vivacious						0.675

### Second-order factor analysis

The six factors were analyzed further. Based on the items included in each factor, the first four items of each factor were subjected to second-order factor analysis. Factors with characteristic roots of greater than one were extracted (Tables 3–6). Analyses were performed using the above-described procedure.

**Table 3:** Factor load matrix of *F*1.

Adjective	*F*11	*F*12	*F*13
Cheerful	0.613		
Optimistic	0.554		
Lively	0.641		
Interesting	0.635		
Passionate	0.601		
Good-hearted		0.611	
Magnanimous		0.635	
Open		0.698	
Forthright		0.687	
Bold		0.595	
Extroverted			0.616
Talkative			0.576
Impulsive			0.710
Excited			0.651

**Table 4:** Factor load matrix of *F*2.

Adjective	*F*21	*F*22	*F*23
Indomitable	0.704		
Tolerant	0.626		
Valiant	0.602		
Aspiring	0.647		
Fortitude	0.660		
Persevering		0.639	
Active		0.682	
Pioneering		0.653	
Eager to excel		0.617	
Tireless			0.631
Hardworking			0.655
Respectful of the profession			0.557
Persistent			0.543

**Table 5:** Factor load matrix of *F*3

Adjective	*F*31	*F*32
Forgiving	0.463	
Sane	0.607	
Flexible	0.611	
Adaptive	0.627	
Self-motivated	0.636	
Team-spirited		0.624
Articulate		0.655
Valuing friendship		0.604
Loyal to friends		0.637
Willing to help others		0.547

**Table 6:** Factor load matrix of *F*4.

Adjective	*F*41	*F*42
Perfectionist	0.636	
Continuously improving	0.570	
Advanced	0.651	
Striving for excellence	0.612	
Seeking to better oneself	0.679	
Independent		0.622
Confident		0.630
Focused		0.681
Opinionated		0.665
Person of character		0.570

A factor analysis was performed on the 17 items of factor *F*1. Three second-order factors were used to explain 61.78% of the total variance: *F*11 (cheerful): cheerful, optimistic, lively, interesting, and passionate; *F*12 (good-hearted): good-hearted, magnanimous, open, forthright, and bold; and *F*13 (extroverted): extroverted, talkative, impulsive, and excited; the three second-order factors of *F*1, respectively, explained 21.87, 20.06, and 19.93% of the variance. Three items, i.e., open-minded, courageous, and frank and outspoken, were eliminated due to the presence of multiple loads (Table 3).

A factor analysis was performed on the 15 items of factor *F*2. Three second-order factors were found to explain 53.25% of the total variance: *F*21 (courageous): indomitable, tolerant, valiant, aspiring, and fortitude; *F*22 (enterprise): persevering, active, pioneering, and eager to excel; and *F*23 (diligent): tireless, hardworking, respectful of the profession, and persistent; the three second-order factors of *F*2, respectively, explained 19.72, 16.96, and 16.56% of the variance. Two items, i.e., dedicated and dauntless, were removed due to the presence of multiple loads (Table 4).

A factor analysis was performed on the 12 items of factor F3. Two second-order factors were found to explain 46.39% of the total variance: F31 (mature): forgiving, sane, flexible, adaptive, and self-motivated; and F32 (group-oriented): team-spirited, articulate, valuing friendship, loyal to friends, and willing to help others; the two second-order factors of F3, respectively, explained 23.87 and 22.52% of the variance(Table 5).

A factor analysis was performed on the 10 items of factor *F*4. Two second-order factors were found to explain 58.16% of the total variance: *F*41(outstanding): perfectionist, continuously improving, advanced, striving for excellence, and seeking to better oneself; and *F*42 (independent): independent, confident, focused, opinionated, and person of character; the two second-order factors of F4, respectively, explained 29.47 and 28.69% of the variance (Table 6).

From the first- and second-order factor analyses, the psychological structure of sports culture was mostly confirmed (Table 7).

**Table 7:** Composition and second-order factor items of the psychological structure of sports culture.

*F*1 Extroversion-activity	*F*2 Diligence-progression	*F*3 Experience	*F*4 Independence-excellence	*F*5 Enjoyment	*F*6 Body culture
F11 cheerful	F21 courageous	F31 mature	F41 outstanding	F51 fun loving	F61 body culture
cheerful	indomitable	forgiving	perfectionist	casual	masculine
optimistic	tolerant	sane	continuously improving	relaxed	agile
lively	valiant	flexible	advanced	fun loving	powerful
interesting	aspiring	adaptive	striving for excellence	joyful	strong
passionate	fortitude	self-motivated	seeking to better oneself	comfortable	vital
*F*12 good-hearted	*F*22 enterprise	*F*32 group-oriented	*F*42 independent		
good-hearted	persevering	team-spirited	independent		
magnanimous	active	articulate	confident		
open	pioneering	valuing friendship	focused		
forthright	eager to excel	loyal to friends	opinionated		
bold		willing to help others	person of character		
*F*13 extroverted	*F*23 diligent				
extroverted	tireless				
talkative	hardworking				
impulsive	respectful of the profession				
excited	persistent				

### Reliability analysis

Cronbach *α* coefficients of the six main factors were found to range from 0.53 to 0.66. The high degree of variance in the adjectives denotes the homogeneous reliability of the structure. Six weeks later, a retest was performed on 93 undergraduate students. The test–retest reliability level was between 0.74 and 0.82 (Table 8).

**Table 8:** Homogeneity and test–retest reliability of the psychological structure of sports culture.

	*F*1	*F*2	*F*3	*F*4	*F*5	*F*6
Cronbach α	0.53	0.55	0.62	0.61	0.65	0.66
Test–retest reliability	0.76	0.74	0.75	0.79	0.81	0.82

## Discussion

The sports culture is a complex, multidimensional phenomenon. The conceptual contents of its structural components lack a common perspective. In other words, different theories are premised on different concepts. Therefore, other structures of sports culture must also be explored through the implementation of other theoretical frameworks and approaches. This paper argues that sports culture, as a social phenomenon, is an integral part of the culture and that its systemic formative factors are sporting values and individual attitudes towards sport and physical activity (Seppnen, 1989). Human physical culture results from the internalizing process of the cultural and educational potential, values, and techniques of the sport, the accumulation of human experience of physical culture and physical activity, and the discovery of individual consciousness in physical culture and physical activity. From this perspective, it is possible to configure the components of sporting culture through the study of psychological structures.

The structure of complex phenomena, which also refers to the human personality, represents a combination of different elements, layers, and certain interactions. The structure of sports culture represents some personality and social values (Hughson, 2009). For example, Korovin (2016) used physical adaptation, cognitive-intellectual, and axiology to construct the components of Professional Physical Culture of Personality(PPCP). The model of 6 dimensions-12 factors identified through this study shows that sports culture is not only a body culture but also a psychological and social culture, demonstrating both the rationality of Apollonian culture and the romance of Dionysian culture, which seeks not only excellence but also health. The spirit of personality and the formation of values is fundamental to all the elements of activity in sports culture because the relationship of human values which runs through its elements, is the standard core of personality, the backbone elements of sports culture, and the determination to the specific expression of the other elements. Also, the sport has a subjective and universal structure that organizes sporting activities for specific competitions and gives them a clear meaning.

The clusters and factors extracted were meaningful and interpretable. However, there was still some limitation in this study. First, the research mainly relied on participants’ self-reported or hypothetical situational responses, and their relevance to actual behavior needs to be replicated (Baumeister et al., 2007). Second, the empirical research on the structure of sports culture is still in its infancy, and the validity of the 6-dimensional 12-factor model developed through a psychological normative process needs to be further expanded and validated. Third, word association is a critical tool for identifying intrinsic values (Angleitner et al., 1990). And vocabulary sorting by two linguistic experts might be subjective. It is important to note that vivid adjectives obtained through everyday conversation can vary greatly, which requires increased vocabulary through other means to reduce variation in future studies. Finally, cross-cultural research should be assessed by using the structural approach and by comparing the position of these factors in the field of research and in the field of social practice.

## Data availability statement

The original contributions presented in the study are included in the article/supplementary material, further inquiries can be directed to the corresponding authors.

## Ethics statement

Ethical review and approval was not required for the study on human participants in accordance with the local legislation and institutional requirements. Written informed consent from the patients/participants or patients/participants legal guardian/next of kin was not required to participate in this study in accordance with the national legislation and the institutional requirements.

## Author contributions

XH and WW were responsible for the design of the study. YG and NY were responsible for data collection and essay writing. All authors contributed to the article and approved the submitted version.

## Funding

This project is financially supported by the 14th Five-Year-Plan Advantageous and Characteristic Disciplines (Groups) of Colleges and Universities in Hubei Province for Exercise and Brain Science from Hubei Provincial Department of Education in China, Major Project for Prosperity of Philosophy and Social Sciences in Hubei Province (21ZD096), Ministry of Education Project of Research of Humanitarian (19YJC890013) and Young and Middle-aged Scientific Research Team of Wuhan Sports University in 2021 (21KT06).

## Conflict of interest

The authors declare that the research was conducted in the absence of any commercial or financial relationships that could be construed as a potential conflict of interest.

## Publisher’s note

All claims expressed in this article are solely those of the authors and do not necessarily represent those of their affiliated organizations, or those of the publisher, the editors and the reviewers. Any product that may be evaluated in this article, or claim that may be made by its manufacturer, is not guaranteed or endorsed by the publisher.
